# Epidermal growth factor or platelet-rich plasma combined with induced membrane technique in the treatment of segmental femur defects: an experimental study

**DOI:** 10.1186/s13018-020-02142-2

**Published:** 2020-12-11

**Authors:** Ökkeş Bilal, Duran Topak, Mustafa Kınaş, Ergül Belge Kurutaş, Betül Kızıldağ, Abdulkadir Yasir Bahar

**Affiliations:** 1 Department of Orthopaedic and Traumatology, Faculty of Medicine, Kahramanmaras Sutcu Imam University, Kahramanmaras, Turkey; 2 Private Bandırma Royal Hospital, Balıkesir, Turkey; 3 Department of Biochemistry, Faculty of Medicine, Kahramanmaras Sutcu Imam University, Kahramanmaras, Turkey; 4 Department of Radiology, Faculty of Medicine, Kahramanmaras Sutcu Imam University, Kahramanmaras, Turkey; 5 Department of Pathology, Faculty of Medicine, Kahramanmaras Sutcu Imam University, Kahramanmaras, Turkey

**Keywords:** Epidermal growth factor (EGF), Platelet-rich plasma (PRP), Masquelet technique, Induced membrane, Experimental study, Femur

## Abstract

**Objective:**

Extensive bone defects remain a therapeutic challenge necessitating alternative surgical approaches with better outcomes. Can increase the effectiveness of PRP or EGF treatment in surgical treatment of large bone defects with Masquelet technique?

Aim of this study examined potential therapeutic benefits of the Masquelet technique with induced membranes in combination with platelet-rich plasma (PRP) or epidermal growth factor (EGF) in a rat model of segmental femur defect.

**Methods:**

Three groups each consisting of 20 Sprague-Dawley rats were defined as follows: EGF group, PRP group, and control group. A femoral bone defect was created and filled with antibiotic embedded polymethyl methacrylate. Half of the animals in each group were sacrificed at week 6 and the pseudo-membranes formed were analyzed. In the remaining half, the cement was removed and the space was filled with autograft. After another 6 weeks, the structures formed were examined radiologically, histologically, and biochemically.

**Results:**

At week 6, both PRP and EGF groups had significantly higher membrane CD31, TGF-beta, and VEGF levels than controls. At week 12, when compared to controls, PRP and EGF groups had significantly higher membrane CD31 levels and the PRP group had significantly higher membrane TGF levels. Regarding bone tissue levels, PRP and EGF groups had significantly higher VEGF levels and the EGF group had significantly higher BMP levels. In addition, PRP and EGF groups had higher radiological scores than controls. However, the two experimental groups did not differ with respect to any parameter tested in this study.

**Conclusion:**

Both PRP and EGF seem to be associated with histological, biochemical, and radiological improvements in experimental rat model of Masquelet technique, warranting in further clinical studies.

**Level of evidence:**

Level 5

## Introduction

Extensive bone defects due to traumatic injury remain a therapeutic challenge in terms of anatomical and functional outcomes. The traditional approach for restoration of bone defects requires extensive surgical intervention involving the use of bone grafting that is associated with donor-site complications and frequent occurrence of morbidity, with no guarantee of complete correction of the defect [1]. Thus, the search for alternative surgical approaches continues. Recently, Masquelet technique has been described as a two-stage treatment strategy for large bone defects that consists of a temporary cement spacer followed by bone grafting [2]. The technique allows reconstruction of extensive diaphysis defects even in the presence of previous radiation exposure or infections.

Platelet-rich plasma (PRP) is a volume of fractionated plasma from patient’s own blood containing platelet concentrate [3]. PRP contains several growth factors that play a major role in tissue repair mechanisms including but not limited to platelet-derived growth factor, transforming growth factor-beta, and vascular endothelial growth factor. As a result of its potential therapeutic effects, PRP has recently gained significant attention as a safe nonsurgical adjunctive treatment modality for osteoarthritis and musculoskeletal repair [4]. Despite the lack of definitive evidence for the therapeutic benefit of PRP in bone healing [5, 6], a multitude of recent experimental and clinical publications have suggested a potential utility [7–13].

As compared to PRP, however, published data on the use of epidermal growth factor (EGF) in orthopedic surgery is very scarce and most information relates to its potential therapeutic benefits in conditions other than bone healing such as wound healing, diabetic foot ulcers, or experimental dentistry [14–18].

In the meta-analysis study of the Masquelet technique performed by Morelli et al., it was shown that bone union occurred in 89.7% of the cases, and the rest did not [19].

This study was undertaken as an initial experimental attempt to assess the feasibility and potential therapeutic benefits of the Masquelet technique with induced membranes in combination with PRP and EGF in a rat model of segmental femur defect. The assessments included comparison of tissue bone morphogenetic protein 2 (BPM-2), transforming growth factor-beta (TGF-beta), and vascular endothelial growth factor (VEGF) levels as markers of bone healing, osteoinduction, and angiogenesis, as well as histopathological and radiological examinations in experimental study groups.

## Materials and methods

### Design and experimental animals

A total of 60 male Sprague-Dawley rats weighing between 400 and 450 g were used for the experimental protocol. Three groups each consisting of 20 rats were defined as follows: EGF group, PRP group, and control group. Standard housing and feeding conditions were provided for the animals. The study protocol was approved by Kahramanmaras Sutcu Imam University Medical Faculty Animal Experimentation Ethics Committee (session: 2014/01, date: 17.04.2014, decision no: 05).

**Fig. 1:**
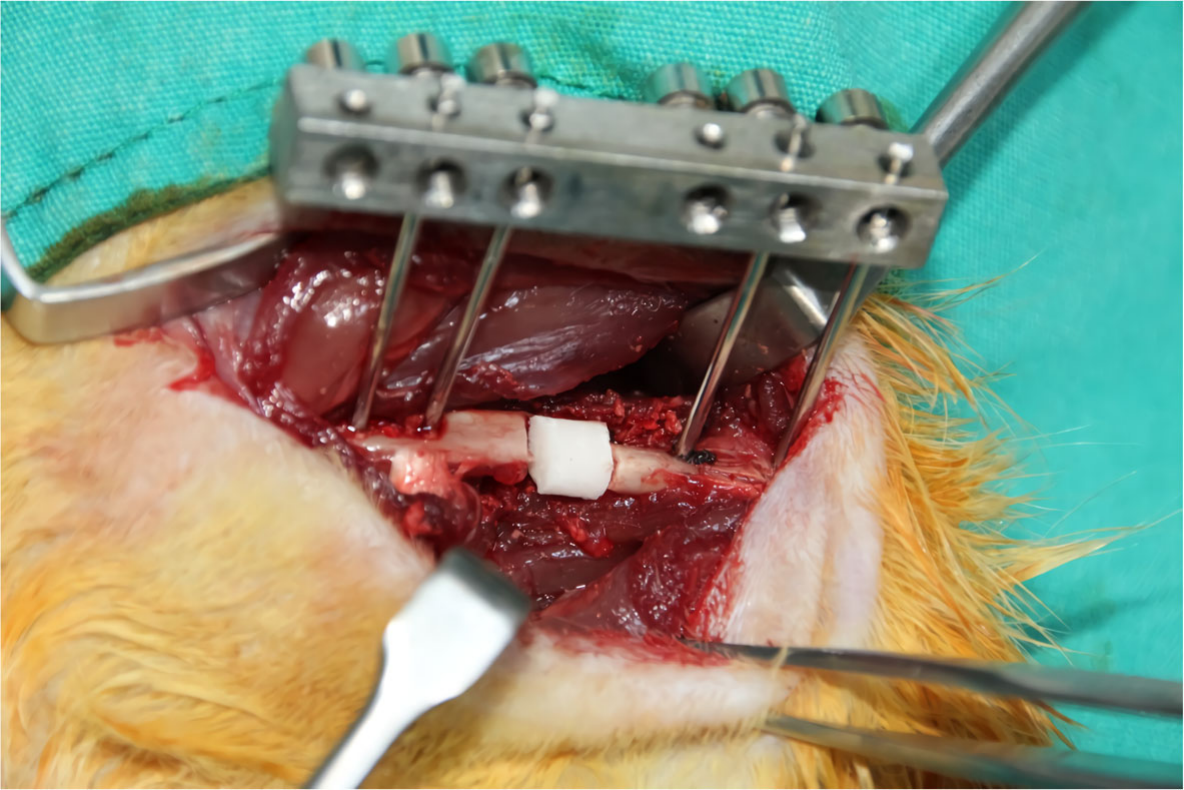
Segment creation and spacer application with antibiotic-embedded cement in rat femur (blue arrow)

A 5-mm bone defect was created and filled with antibiotic embedded polymethyl methacrylate followed by the fixation of the femur using external fixators. Figure 1 shows the defect model. Half of the animals in each group were sacrificed at week 6 and the pseudo-membranes formed were prepared for study analyses. In the remaining half of the groups, the cement was removed and the resultant space was filled with autograft obtained from the tail of each animal. After 6 weeks, the bony structures formed were examined radiologically, in addition to histological and biochemical assessments.

### Procedures (first step)

Prior to surgery, each animal received a single 0.1 mg/kg intramuscular dose of cefazolin sodium (Cefozin, Bilim Ilac, Turkey) prophylactically. For surgery, anesthesia was provided with intramuscular ketamine HCl 200 mg/kg (Ketalar®Eczacibasi, Turkey) and xylazine 1 mg/kg (RompunR, Bayer, Turkey). Right hind legs were shaved and covered with sterile covers after disinfection with Betadine (polyvidone iodine solution). The skin and subcutaneous tissues were dissected in accordance with surgical principles to expose the femur of rats. A 5-mm bony defect was created using a 1-mm drill tip and osteotome, and then it was filled with antibiotic embedded polymethyl methacrylate (Cemex Genta ID Green TECRES® Italy). Femur was fixated using external fixators, and the skin was sutured using 4/Ethilon nylon monofilament suture material. EGF and PRP were injected into the wound area.

Rats in the epidermal growth factor group received, epidermal growth factor was injected into bone defect area at a dose of 25 μg/ml once a week for 3 weeks (HEBERPROT-P® 75 μg, Hasbiotech Ilac San. ve Tic. A.S. Turkey). The blood obtained from the rats in the PRP group was used to prepare platelet-rich plasma using a MAGELLAN® Autologous Platelet Separator System (Arteriocyte Medical Systems USA). It was injected into the bone defect area once a week for 3 weeks volumes of 1 ml. Controls received weekly injections of 1 ml of physiological saline around the cement. Half of the rats were sacrificed at the end of 6 weeks using high dose pentobarbital anesthesia and the pseudo-membranes forming around the space filled with cement were extracted. Each membrane sample was divided into two halves, one being fixed in formaldehyde for histopathological assessments, and the other kept at −40 °C for biochemical analyses. Three animals in the EGF and one in the control group died and were therefore excluded from the analyses.

### Procedures (second step)

In the remaining rats in study groups, the same procedures as described in step one were used to expose the femurs. The pseudo-membranes formed were carefully and longitudinally opened and autogenic bone grafts retrieved from the tail of each animal were placed into the resulting space. The operation site was sutured.

Six weeks after these procedures, anteroposterior and lateral X-rays and CT images were obtained for radiological assessments. The rats were sacrificed with high dose pentobarbital anesthesia, and the pseudo-membranes around the defect into which grafts were placed and bone tissue were removed and cut into two equal pieces, one being fixed in formaldehyde for histopathological assessments, and the other kept at −40 °C for biochemical analyses. At this stage, 3, 4, and 2 animals died in PRP, EGF, and control groups, respectively, and were therefore excluded from the analyses. Paracetamol was administered to rats in appropriate doses as a pain reliever during the study.

### Biochemical assessments

Rat tissue samples were placed into ice as soon as they were removed, blotted, and weighed. Homogenization for 15 to 20 min was performed in a homogenizer with ice boxes containing 1 g of tissue per 5 volume of cold 1.15% KCl (weight/volume). Then, the homogenate was centrifuged for 30 min at 14000 rpm at +4 °C, and the biochemical analyses were done on the supernatant. ELISA double-sandwich methodology was used for BMP-2 (pg/mL), TGF-beta (ng/mL), and VEGF (pg/mL) assessments and BMP-2, VEGF and TGF-beta levels in the tissue samples were measured in duplicate using commercially available solid-phase sandwich enzyme-linked immunosorbent assay (ELISA) kits (MyBioSource Company, USA) according to the manufacturer’s protocol.

### Histopathological assessments

**Fig. 2:**
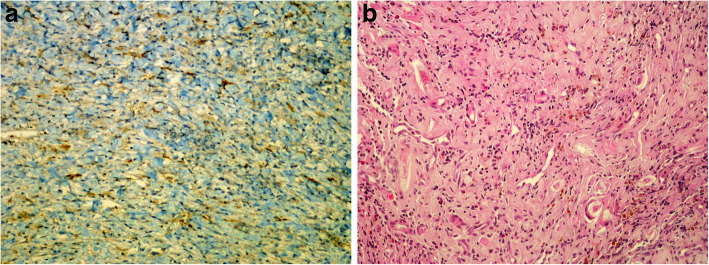
Pathological images of fracture healing in experimental cases. **a** Microscopic image of prepatellar stained with CD 31 at ×200 magnification (brown areas indicate endothelial cells stained with CD 31). **b** Microscopic image of prepatellar stained with hematoxylin eosin at ×200 magnification (areas of inflammatory granulation tissue was shown)

The samples were fixed for 24 h in 10% buffered formaldehyde and then were embedded. After obtaining appropriate cross-sections from paraffin blocks, they were stained with hematoxylin and eosin (HE) and were examined under a light microscope (Olympus-BX53). In order to perform a histochemical assessment of the neovascularization of the pseudo-membrane, granulation tissue was identified in HE stained cross sections. A histopathologist, unaware of the study procedures, was included in the study. Based on its high specificity and sensitivity as an endothelial marker, CD31 (platelet endothelial cell adhesion molecule—PECAM1) was chosen as a marker of endothelial proliferation and neovascularization, and was applied immune histochemically to the areas of granulation identified with HE staining. Cross-sections of 3-micron thickness were stained with CD31 using a “LEICA BENCHMARK XT” immunohistochemistry device and were assessed using a light microscope with quantification of vascular structures (capillaries as well as immature vascular structures) in each 1 mm^2^ at ×40 magnification power (Fig. 2).

### Radiological assessments

**Fig. 3:**
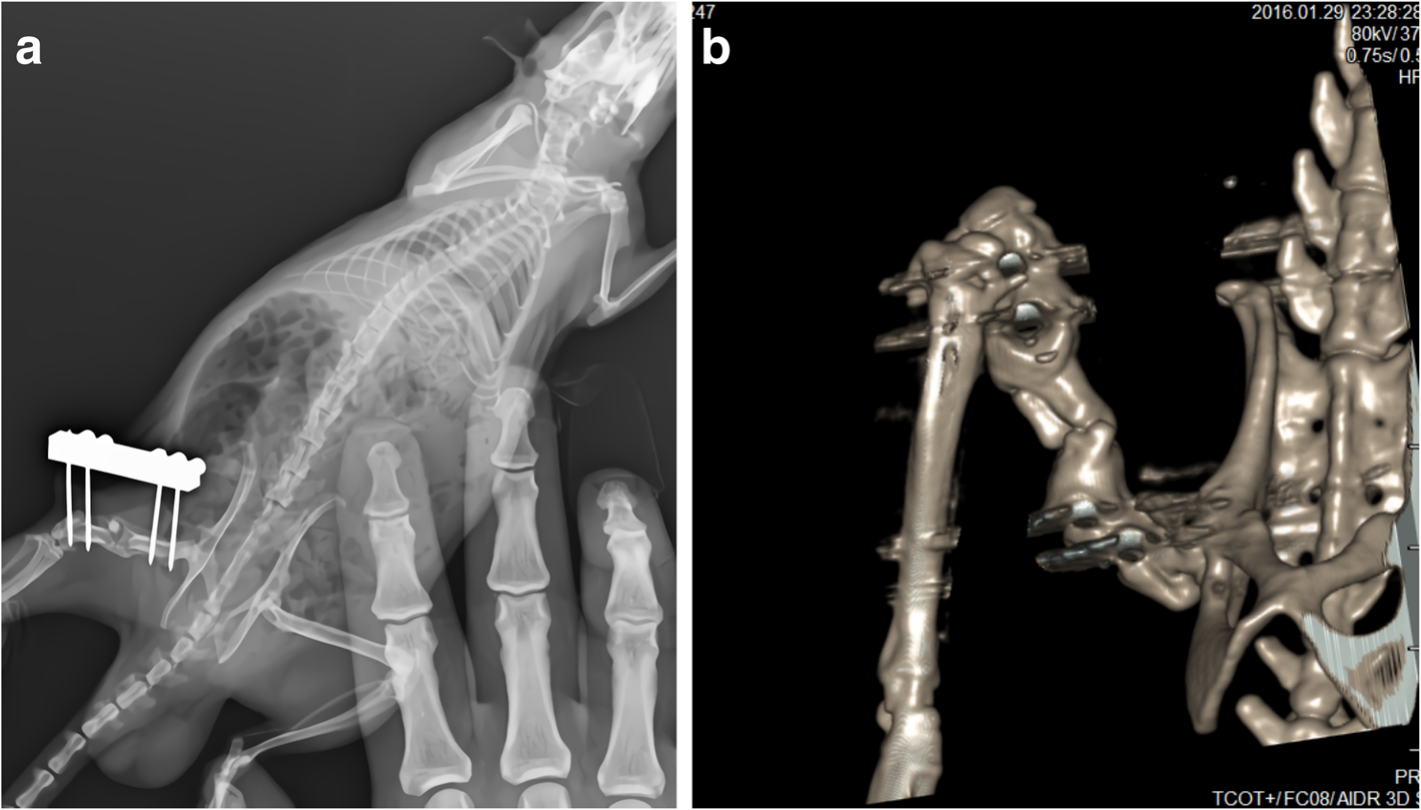
Radiological image of experimental cases. **a** Radiographic image of the case in the PRP group after 6 weeks (Lane-Sandhu score 4). **b** Computed tomography image of the case in the PRP group after 6 weeks (Lane-Sandhu score 4)

In each rat, antero-posterior and lateral X-rays as well as computed tomography images (Toshiba Alexon 16 multi-slice) were obtained (Fig. 3). A radiologist blinded to the study procedures used a CT version of Lane-Sandhu scoring system for image scoring as follows: 0, no callus, clear fracture line; 1, 25% callus tissue, the fracture line can be clearly observed; 2, 50% callus tissue, blurred fracture line; 3, 75% callus tissue, fracture line barely visible; 4, 100% callus tissue, fracture line cannot be seen [19].

### Statistical analyses

Statistical Package for Social Sciences (SPSS) version 21 was used for the analysis of data. Normality was tested using Shaphiro-Wilk test and graphical methods. Kruskal-Wallis test or analysis of variance (ANOVA) was used to test intergroup differences, and built-in post hoc test for Kruskal-Wallis or Tukey HSD was used for pairwise comparisons, respectively. A *p* value smaller than 0.05 was considered the indication for statistical significance.

## Results

Table 1 shows the comparison of the groups with respect to study assessments, at week 6 and week 12.

**Table 1:** Comparison of the groups with regard to histological, biochemical, and radiological study assessments

	Group 1 (PRP)	Group 2 (EGF)	Group 3 (controls)	***p***
**Assessments at week 6** (mean ± SD)				
Histological CD31 assessment (counts/mm^2^)	143.7 ± 8.2	151.1 ± 9.2	125.2 ± 13.3	< 0.001
Membrane TGF level (ng/mL)	246.4 ± 54.3	257.7 ± 55.2	141.8 ± 20.8	< 0.001
Membrane VEGF level (pg/mL)	842.0 ± 98.5	754.8 ± 118.7	430.5 ± 92.2	< 0.001
**Assessments at week 12** (mean ± SD)				
Histological CD31 assessment (counts/mm^2^)	137.3 ± 17.3	138.1 ± 14.0	99.6 ± 25.5	0.003
Membrane TGF level (ng/mL)	397.4 ± 44.4	379.3 ± 63.3	326.4 ± 42.0	0.032
Membrane VEGF level (pg/mL)	318.0 ± 95.2	273.0 ± 72.1	228.9 ± 44.9	0.086
Bone VEGF level (pg/mL)	192.2 ± 6.7	198.1 ± 5.7	176.3 ± 7.2	0.003
Bone BMP-2 level (pg/mL)	191.9 ± 8.4	237.6 ± 37.3	117.1 ± 43.0	0.012
**Lane score** (median, range)				
Anteroposterior	4 (3-4)	3 (2-4)	1 (1-2)	0.015
Lateral	4 (2-4)	3.5 (2-4)	1 (1-2)	0.026
CT	3 (2-4)	3 (2-3)	1 (1-2)	0.026
3D	3.5 (2-4)	4 (3-4)	1.5 (1-3)	0.044

Abbreviations: *PRP* platelet rich plasma, *EGF* epidermal growth factor, *CD 31* cluster of differentiation 31, *TGF-beta* transforming growth factor-beta, *BPM-2* bone morphogenetic protein 2, *VEGF* vascular endothelial growth factor, *CT* computed tomography, *3D* three-dimensional

### Membrane tissue assessments at week 6

At week 6, the PRP group had significantly higher membrane CD31 (*p* = 0.002), TGF (*p* < 0.001), and VEGF (*p* < 0.001) levels than controls. Similarly, the EGF group had significantly higher membrane CD31, TGF-beta, and VEGF levels than controls (*p* < 0.001 for all three comparisons). However, the two groups did not differ with regard to any of these three parameters.

### Biochemical, histological, and radiological assessments at week 12

At week 12, PRP (*p* < 0.014) and EGF (*p* < 0.008) groups had significantly higher membrane CD31 levels than controls, with no significant difference between the former two groups. PRP group had significantly higher membrane TGF-beta levels than controls (*p* = 0.032); however, no other pairwise difference reached significance.

Regarding bone tissue levels, PRP (*p* < 0.019) and EGF (*p* < 0.003) groups had significantly higher VEGF levels than controls, without any significant difference between the former two groups. On the other hand, the EGF group had significantly higher bone BMP levels than controls (*p* = 0.010), with no other significant pairwise differences.

### Lane scores at week 12

PRP group had significantly higher AP (*p* = 0.013), lateral (*p* = 0.040), and CT scores (*p* = 0.040) than controls, whereas no other pairwise difference was evident regarding these parameters. On the other hand, the EGF group had significantly higher 3D scores than controls (*p* = 0.047), representing the only significant pairwise difference for this parameter.

## Discussion

This study examining the effect of two therapeutic approaches, i.e., PRP and EGF, on bone healing when used as an adjunct to the Masquelet technique for the management extensive bone defects has provided promising results in terms of a potential therapeutic benefit. Accordingly, both PRP and EGF were associated with improved biochemical and histological outcomes, both at 6- and 12-week assessment timepoints, as reflected by higher membrane/bone tissue CD31, TGF-beta, and VEGF levels in PRP and EGF groups. In addition, these adjunctive treatments to the Masquelet technique resulted in radiological improvements in AP lateral X-ray and CT scores (for PRP) and 3D scores (EGF). Of these two approaches, PRP has previously been subject to extensive research regarding its therapeutic utility in orthopedic procedures, while literature data on EGF is rather scarce. Furthermore, to the best of our knowledge, no previous studies have examined these two agents in combination with the Masquelet technique in such an experimental setting.

PRP has been generally reviewed as “a promising therapeutic approach for future regenerative treatments” [4] and a great majority of the preclinical studies on the treatment of bone defects support the use of PRP [5]. Specifically, positive results have been reported in rabbit ulnar defects [20], rat femoral fractures [11], as well as in rabbit tibia shaft fractures [21]. In humans, clinical reports on the healing rate of long bone non-union fractures have also indicated positive outcomes in terms of cure rate, healing duration, and limb shortening [8, 9]. Despite these initial promising results, it has been underscored that the evidence to support the routine use of this intervention in clinical practice is currently insufficient [10].

In contrast with PRP, research on the possible therapeutic effects of epidermal growth factor (EGF) in tissue healing is mainly limited to the field of experimental dentistry [15, 17], chronic diabetic foot ulcers [14], or other types of soft tissue pathologies including chronic radiation ulcers [18]. On the other hand, EGF receptors are known to play a role in endochondral bone formation [22], justifying research assessing its potential effects in bone healing. For instance, loss of epidermal growth factor receptor (EGFR) activity was found to alter the development of the growth plate, impair endochondrial ossification, and retard the growth in a mice model [22]. Furthermore, administration of EGF in liposomes resulted in a faster recovery of injured alveoli of rats after the extraction of the maxillary second molar teeth, providing protective effects against early absorption and degradation [17]. In a case report exploring the potential benefits of recombinant epidermal growth factor in a patient with radiation-induced chronic wound, successful healing within 16 weeks was reported after the failure of flap surgery and conventional treatments given over a 3=year period [18]. Similar enhancement of wound healing leading to thicker epidermal and dermal layers was also reported with the use of decellularized scaffolds loaded with epidermal growth factor in an experimental model of wound healing [16]. Overall, our results are in concordance with the abovementioned findings as reflected by improved biochemical, histopathological, as well as radiological outcomes in rats receiving additional EGF treatment as a part of the Masquelet technique for segmental bone defects.

Production of growth factors (VEGF, TGF-beta) as well as osteoinductive factors (BMP-2) has been previously documented in the induced membranes in a rabbit model of the Masquelet technique [23]. Furthermore, platelets are known to contain alpha-granules that are rich in a number of growth factors including platelet-derived growth factor, transforming growth factor-beta, insulin-like growth factor, vascular endothelial growth factor, and epidermal growth factor, which are of major significance in tissue repair mechanisms [4].

Thus, our results corroborate previous studies and provide further evidence for the first time that PRP and EGF have the potential to be used as an adjunct for accelerated healing in the Masquelet technique for the management of extensive bone defects. However, the small sample size is certainly a limitation of our study. Further studies may confirm our findings for each of these agents and may possibly explore the utility of the combined use of these two agents for potential synergistic therapeutic effects.

A limitation of the current study could be considered to be the low sample size in the groups, but the number was appropriate according to the power analysis calculation. Although CD31 was found to be sufficient for vascularization analysis in rats, other vascularization indicators such as vWF could be added.

## Conclusions

Masquelet technique is an effective surgical method that can be applied easily in the restoration of large bone defects. In the Masquelet technique, pseudomembrane formation is the main factor in the restoration of bone defect. The addition of EGF or PRP to the Masquelet technique has been shown to increase vascularization in the pseudomembrane.

Histopathological and biochemical healing displayed by the addition of EGF or PRP to the Masquelet technique in the restoration of large bone defects also provided a radiologically significant improvement.

Both PRP and EGF were associated with improved histological, biochemical, and radiological improvements in this experimental rat model of the Masquelet technique. These promising observations may be confirmed in further clinical studies involving humans.

## Data Availability

The data and materials of patients participating in this study are available to us and will be provided by us upon request.
